# Spatiotemporal progression of metastatic breast cancer: a Markov chain model highlighting the role of early metastatic sites

**DOI:** 10.1038/npjbcancer.2015.18

**Published:** 2015-10-21

**Authors:** Paul K Newton, Jeremy Mason, Neethi Venkatappa, Maxine S Jochelson, Brian Hurt, Jorge Nieva, Elizabeth Comen, Larry Norton, Peter Kuhn

**Affiliations:** 1 Department of Aerospace and Mechanical Engineering, University of Southern California, Los Angeles, CA, USA; 2 Department of Mathematics, University of Southern California, Los Angeles, CA, USA; 3 Department of Biological Sciences, Dornsife College of Letters, Arts and Sciences, University of Southern California, Los Angeles, CA, USA; 4 Memorial Sloan Kettering Cancer Center, New York, NY, USA; 5 University of Colorado School of Medicine, Aurora, CO, USA; 6 Norris Comprehensive Cancer Center, Keck School of Medicine, University of Southern California, Los Angeles, CA, USA

## Abstract

**Background:**

Cancer cell migration patterns are critical for understanding metastases and clinical evolution. Breast cancer spreads from one organ system to another via hematogenous and lymphatic routes. Although patterns of spread may superficially seem random and unpredictable, we explored the possibility that this is not the case.

**Aims:**

Develop a Markov based model of breast cancer progression that has predictive capability.

**Methods:**

On the basis of a longitudinal data set of 446 breast cancer patients, we created a Markov chain model of metastasis that describes the probabilities of metastasis occurring at a given anatomic site together with the probability of spread to additional sites. Progression is modeled as a random walk on a directed graph, where nodes represent anatomical sites where tumors can develop.

**Results:**

We quantify how survival depends on the location of the first metastatic site for different patient subcategories. In addition, we classify metastatic sites as “sponges” or “spreaders” with implications regarding anatomical pathway prediction and long-term survival. As metastatic tumors to the bone (main spreader) are most prominent, we focus in more detail on differences between groups of patients who form subsequent metastases to the lung as compared with the liver.

**Conclusions:**

We have found that spatiotemporal patterns of metastatic spread in breast cancer are neither random nor unpredictable. Furthermore, the novel concept of classifying organ sites as sponges or spreaders may motivate experiments seeking a biological basis for these phenomena and allow us to quantify the potential consequences of therapeutic targeting of sites in the oligometastatic setting and shed light on organotropic aspects of the disease.

## Introduction

It is widely appreciated that cancer is a multifaceted disease comprised of distinct biochemical, biomechanical, molecular, age, gender, race, and environmental factors, all of which contribute directly or indirectly to uncontrolled cell growth, survival, motility, dissemination, and colonization,^1–5^ which in turn effect long-term survival of patients.^6^ The complex interplay of all of these factors is poorly understood, which hinders our ability to accurately predict and optimally influence outcomes throughout the course of disease progression. As breast cancer spreads from one organ to another via hematogenous and lymphatic routes, cell migration patterns are critical for understanding metastasis and clinical evolution, but these patterns are commonly dismissed as unpredictable in the absence of detailed clinical and patient-specific contextual information. As a consequence, comprehensive quantitative statistical forecasting tools to aid in medical decision making have been slower to develop than in other fields, such as financial forecasting and weather prediction.^7^ For breast cancer, the main prognostic factors in current use include tumor size, patient age, lymph node status, histologic type of tumor, pathologic grade, and hormone-receptor status, and when available, genetic profiling can also be used effectively.^8–19^ But all are based (typically) on a single snapshot of patient information in time and mostly obtained only at the primary tumor location when clinically detectable, hence have limited predictive power with respect to forecasting of disease progression and survival. In other forecasting settings (e.g., weather prediction), it is widely appreciated that collecting data at multiple spatial locations and at multiple time points gives far superior forecasting capability^7^ (even if at lower resolution than a single site) as from these, one is able to obtain estimates of time derivatives (velocities), and spatial gradients, facilitating better estimates not just of the current localized state, but the future distributed state.

In this paper, we explore the possibility that although breast cancer progression in individuals where little additional clinical information is known can be viewed as unpredictable, metastasis patterns assembled over populations of patients that incorporate both temporal and spatial information can be used as a firm basis for predictive modeling and provides an essential step in developing computer-assisted forecasting tools. One of the simplest and most effective dynamical modeling assumptions used in this paper is the Markov assumption that progression from one anatomical location to another proceeds as a weighted random walk on a directed graph, with no history dependence other than the fact that the tumor initiates in the breast. Although not exact, the Markov assumption has been used effectively for a lung cancer data set.^20–23^

Formed by the longitudinal data set of 446 breast cancer patients from Memorial Sloan Kettering Cancer Center assembled over a 25-year period, the Markov transition probabilities from site to site are estimated for each of the groups estrogen receptor (ER)+/human epidermal growth factor receptor 2 (HER2)−, ER−/HER2−, and HER2+. We show that survival depends on the location and characteristics of the first metastatic site to which the disease spreads. In fact, the data show that survival characteristics that use this dynamical and spatial information are potentially as predictive as the ER/HER2 status of a patient. Stated differently, we use information not only on static characteristics of the primary tumor taken as a snapshot in time but also dynamical information on where the disease is spreading, and associated characteristics of the first metastatic site. The location and character of this first site, in turn, have important consequences on the locations and time sequence of subsequent metastatic sites, influencing timescales of disease progression and survival. The full panel of spatiotemporal diagrams and models for each subgroup is available for further study on the interactive website. Additional information associated with treatment scenarios is also available.

## Materials and Methods

### Description of data set

The time-resolved data contain annotated clinical information on 446 patients from the time of their initial diagnosis of breast cancer between 1975 and 2009 at Memorial Sloan Kettering Cancer Center. Notably, the majority of patients were originally diagnosed after 1990, with only 2 patients diagnosed initially in 1975 and 1979, and 25 patients diagnosed in the 1980s. None of the patients had evidence of metastatic disease at the time of diagnosis; all of the patients eventually developed metastatic disease. For each patient, the database contains all clinical and demographic information on the patient from the date of their diagnosis and subsequent development of metastatic disease over time. For each patient, metastatic disease is noted at the time of diagnosis of metastatic disease, usually first detected by positron emission tomography imaging and confirmed by biopsy. Patients were then followed with serial positron emission tomography and/or computed tomography imaging and physical exams. Physical exams were usually done between 1- and 3-month intervals. Imaging was usually done at 3-month intervals. At the time of new metastatic development, site(s) of disease and the date were noted. The treatment rendered and any other pertinent clinical and demographic information is available on each patient. All the relevant information regarding each patient’s original breast cancer diagnosis, including date of pathology report, type of breast cancer, and oncologic and surgical treatment rendered at original diagnosis is noted. All of the information on the treatment rendered throughout metastatic disease course is documented. The date of last follow-up and whether patients are alive or deceased is also noted. Of the 446 patients, 173 patients were alive as of 5 January 2013 and 273 had expired.

### Metastatic progression diagrams

**Figure 1:**
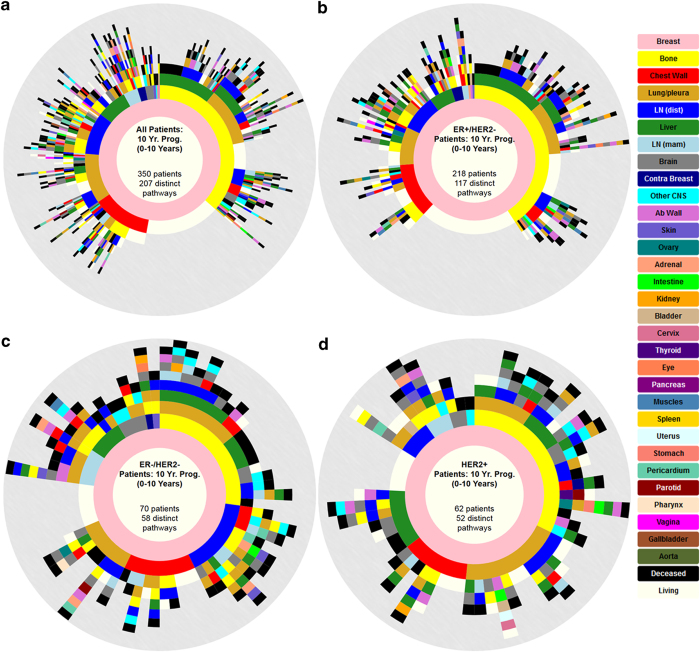
Spatiotemporal progression diagram over a 10-year period of subsets of breast cancer patients. The innermost to outermost rings show progression patterns of primary breast cancer patients (pink ring) and formation of metastases (subsequent rings). Circular arc length of each sector represents the percentage of patients with a metastatic tumor in that location. Bone (yellow) is the most common first metastatic site (first ring outside pink). (**a**) All Patients, (**b**) ER+/HER2−, (**c**) ER−/HER2−, and (**d**) HER2+.

Longitudinal data can be organized most usefully in the form of ring diagrams, as shown in Figure 1a for the entire aggregated data set. Disease progression proceeds from the inner pink ring (primary breast tumor) outward, with each ring representing a subsequent metastatic tumor, color coded according to anatomical site, with a sector size representing the percentage of patients with tumors at that location. The first ring out from the inner ring in Figure 1a shows that bone is the most prominent first metastatic site, in roughly 35% of the patients. The progression of each of the 350 patients proceeds along a ray. The diagram summarizes the complete pathway history (207 distinct pathways) associated with this group of patients tracked over the duration of 10 years. From this data, we can compute the probability of disease “transition” from one anatomical site to any of the others, based on the statistical information contained in the diagrams. This allows us to estimate the entries of the Markov transition matrix associated with disease progression, both in bulk, and for subgroups, which we describe next.

### Markov chains

A Markov chain dynamical system is a discrete-time stochastic process:

$${\vec{v}}_{k+1}={\vec{v}}_{k}A,\;(k=0,1,2,\ldots )$$

where *A* is an nxn transition matrix and ${\vec{v}}_{k}$ is a state vector whose entries indicate the probability of a metastatic tumor developing at each of the *n* anatomical sites, at time step *k*. The time step *k* represents spread from one site to the next in a patient, which can be calibrated with data. The initial state vector in our model is given by ${\vec{v}}_{0}=(1,0,0,0,\ldots )$, where the first entry corresponds to the breast location, indicating that initially there is a tumor located in the breast with probability 1, and no other metastatic tumors at the other locations. The transition matrix *A* has entries whose rows sum to one (corresponding to the fact that they represent probabilities of transition and hence must sum to one), and the *ij*th entry, *a*_*ij*_, indicates the probability of metastatic disease spreading from site “i” to site “j”. We refer the reader to Norris^24^ for a comprehensive introduction to Markov chains and refs 20–23 for recent applications of Markov modeling in the context of lung cancer metastasis. As the longitudinal data are relatively time resolved over long periods, the entries of the transition matrix are obtained in a straightforward empirical way by simple denumeration of disease progression events from one anatomical site to the next in each of the patients in a cohort (see ref. 25 for more general discussions). For example, in tracking a cohort of 100 patients with a primary breast tumor only, if 36 of them subsequently develop their first metastatic tumor in the bone, then the transition probability from breast to bone, obtained empirically, would be 0.36 for this cohort. Note that this number should be interpreted as an estimate based on the length of time the cohort is being followed. In a similar way, by simple denumeration of the distinct metastatic transitions from site to site that each patient follows, we can estimate the Markov transition probabilities from any given site to any other site to create the Markov transition matrix *A*, which drives our model dynamics.

## Results

### Ten-year progression pathways

**Figure 2:**
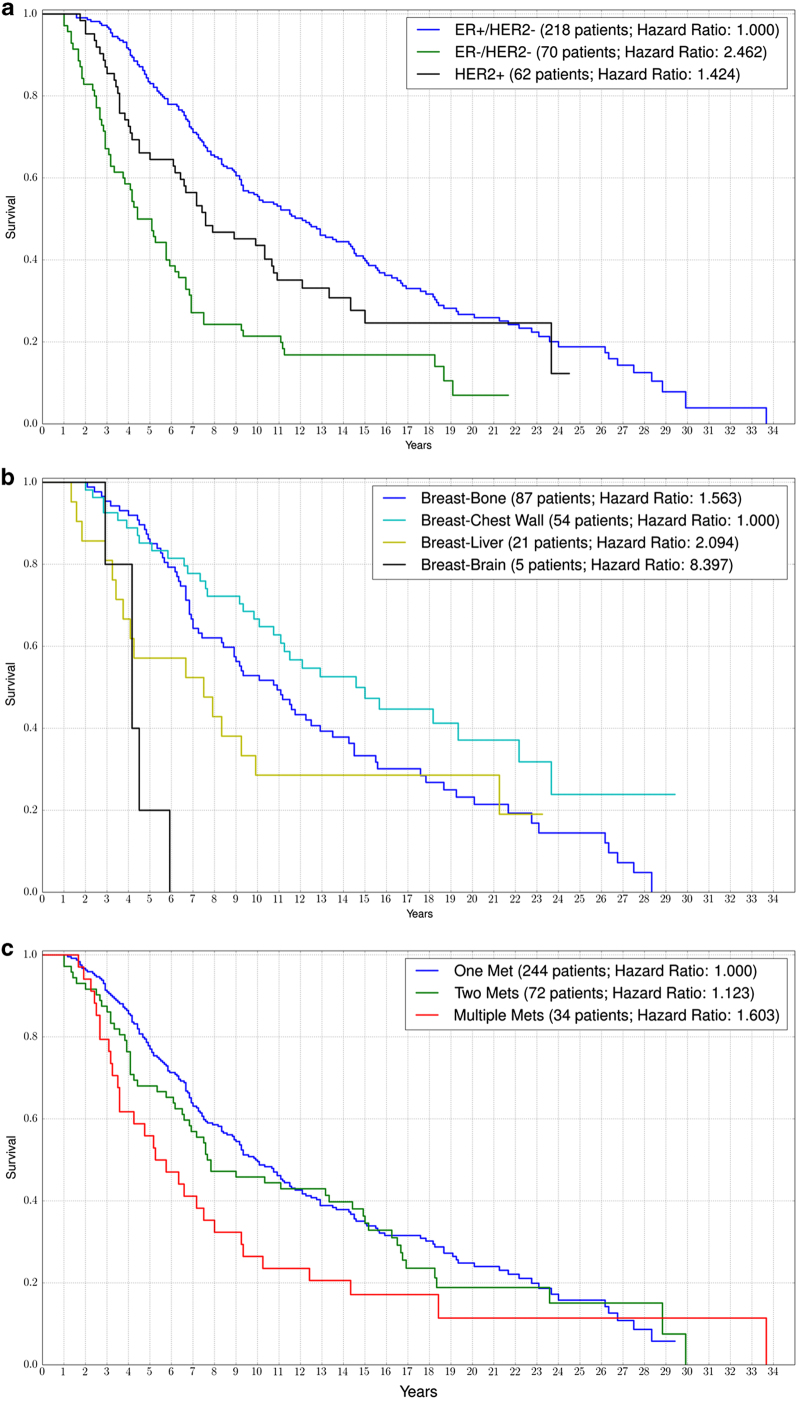
Kaplan–Meier curves showing the survival of breast cancer patients when they initially have no evidence of metastasis to when they progress through their metastatic disease. (**a**) Comparison of ER+/HER2−, ER−/HER2−, and HER2+ patients, (**b**) patients with a solitary first metastatic site at bone, chest wall, liver, or brain, and (**c**) subsets of patients with different numbers of first relapse metastases.

The panels in Figure 1a show ring diagrams associated with 10-year progression representing all patients whom we have a minimum of 10 years of continuous data on starting at the time of diagnosis. These include patients that were enrolled in the study for more than 10 years and those that were expired before the 10-year mark (as we know their metastatic progression after their death date). For the remainder of the paper, we will only focus on those patients that qualify for the 10-year study. The 10-year window was chosen as a balance between having enough patients for statistical significance (i.e., not too long), yet long enough so that significant progression occurred in the cohort under study. The website http://kuhn.usc.edu/breast_cancer/ allows for interactive viewing of these diagrams for both shorter and longer windows. Figure 1a shows the pathways of the 350 eligible patients all grouped together over the 10-year window. For this group, bone metastases are the most prominent first metastatic location, occurring in roughly 35% of the patients being followed. In Figure 2b–d we break the group down into subcategories. Figure 1b shows the ER+/HER2− subgroup (218 patients), where bone metastasis occurs first in roughly 40% of all patients. Figure 1c shows the ER−/HER2− subgroup (70 patients), where bone metastases occurs in a little over 25% of the patients, and Figure 1d shows the HER2+ subgroup (62 patients) with ~33% of patients relapsing first at the bone site. Further examination of the sector sizes in the first metastatic ring shows that the second most common first relapse site differs among the subgroups. For the ER+/HER2− and the ER−/HER2−groups, distant lymph nodes are the second most common first metastatic site, whereas for the HER2+ subgroup, the second most common first relapse site is lung/pleura, followed by chest wall. The diagrams can be viewed from year to year as gif files on the interactive website, giving a dynamic view of the disease as it progresses from the central pink ring outward.

It should be noted that death is a relatively uncommon outcome for a patient with a metastasis to a single organ site, occurring in only 33.33% of patients in the overall cohort. Though it was notably more common in the HER2+ subgroup of patients with liver metastasis, and affected 100% of that subgroup. As expected, the largest number of long-term survivors is recognized as ER+/HER2− patients with bone-only metastasis, with 91.67% of these patients remaining alive during the median 10 years of follow-up. Unexpectedly, we also identified that patients with their first site of metastasis to lung-only metastasis had a favorable percentage of patients going on to be long-term survivors with 68.97% of this subgroup surviving to the end of the 10-year study period. Of note, the majority of the patients with single lung metastasis as their first presentation of metastasis had ER+/HER2− disease, only 5 of the patients with a single lung metastasis at first presentation had triple-negative disease. Interestingly, while many of these patients had lung biopsies to confirm metastatic breast cancer as opposed to a primary lung cancer, they did not routinely undergo resection of the lung metastasis or radiation to the lung. Long-term survivors were also identified in 60.00% of patients with isolated liver metastasis.

### Kaplan–Meier curves

Figure 2 shows Kaplan–Meier survival curves associated with the 10-year cohort that we track. Figure 2a shows survival curves based on the three subgroups ER+/HER2− (218 patients), ER−/HER2− (70 patients), and HER2+ (62 patients). Note that the ER+/HER2− subgroup and the HER2+ subgroup show better survival trends than the ER−/HER2− group.

Survival times are measured from the time of initial breast cancer diagnosis and not from the time of the development of metastatic disease. Patients were treated with Memorial Sloan Kettering Cancer Center standard of care or appropriate clinical trial-based therapy depending on physician recommendations and patient preferences. Of the HER2+ patients, 87.10% received trastuzumab-based therapy at some point during their disease course. HER2+ patients diagnosed before 15 May 2005 did not routinely receive trastuzumab in the adjuvant setting.^26^ After this date, most patients did receive herceptin in the adjuvant setting, which was noted to significantly improve the outcomes for HER2+ breast cancer patients. In the metastatic setting, the Food and Drug Administration originally approved trastuzumab in September of 1998 (ref. 27). All of the ER+ patients received some form of endocrine therapy during the course of their treatment (i.e.,—tamoxifen, aromatase inhibitor, faslodex).

Figure 2b shows survival trends based not on these subgroups, but on groupings associated with the location of the first metastatic site. We focus on four main metastatic sites being bone (87 patients), chest wall (54 patients), liver (21 patients), and brain (5 patients). The patients with first mets to the chest wall have the best prognosis, whereas those with first mets to the brain have the poorest prognosis. Although the 5-year survival of patients with a bone first met is equal to those with a chest wall met (around 85%), the 10-year survival is much worse for chest wall patients as compared with bone patients (50% vs. 70%, respectively). Patients with first mets at the liver have a poor 10-year survival rate (~30%).

Definition of a metastasis was based on global clinical evaluation, which included imaging results, physical examination, and in many cases, biopsy. However, tissue confirmation of a metastatic site was not required for the purposes of the model.

**Figure 3:**
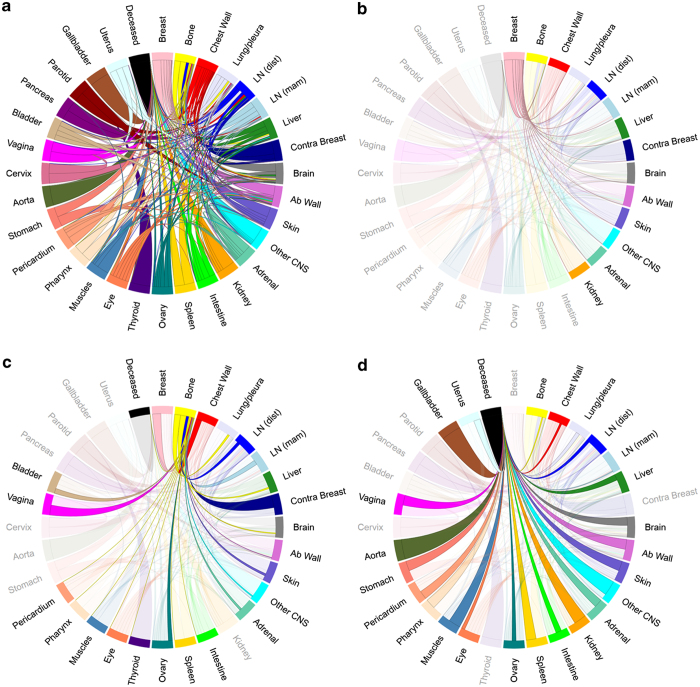
Markov chain networks of metastatic breast cancer shown as circular chord diagrams. Chord widths at their respective starting locations represent one-step transition probabilities between two sites. Primary breast cancer is located on top with metastatic sites ordered clockwise in decreasing order according to transition probability from primary. (**a**) All patients’ network, (**b**) all patients’ network highlighting paths connected to the breast, (**c**) all patients’ network highlighting paths connected to the bone, and (**d**) all patients’ network highlighting paths connected to deceased.

A comparison of Figure 3a, b generally shows that groupings associated with first metastatic location (Figure 2b) gives at least as good an indicator of survival as groupings associated with ER/HER2 status. Figure 2c shows survival of patients with multiple early metastases to various sites. Poorest survival are those with multiple (more than two) first metastases, while much better survival characteristics are associated with those patients with first metastases that are solitary.

The hazard rate of a Kaplan–Meier curve indicates the rate at which survival probability is decreasing within the population being studied. Computing the hazard ratio between two survival curves is a good measure of how much better or worse a certain subgroup is doing compared with another. For example, if group A dies at twice the rate of group B, then the hazard ratio would be 2.

### Markov chain networks and spreader–sponge diagrams

Figure 3 shows the Markov diagrams whose transition values are obtained from the data depicted in the ring diagrams of Figure 1a. The breast site is listed at 12:00 in these diagrams, followed in clockwise decreasing order by the most likely first metastatic sites from the breast. Figure 3a shows the full network diagram clearly depicting the systemic interconnectedness of the anatomical sites throughout the course of the disease. In these diagrams we also use “deceased” as one of the states in the model and list it in the last position. The thickness of the paths leaving each of the sites indicates the proportion of transitions from that site to the receiving site. To clarify this further, we show the outgoing paths from breast (Figure 3b) and bone (Figure 3c). In Figure 3d we show the paths incoming to the deceased site.

**Figure 4:**
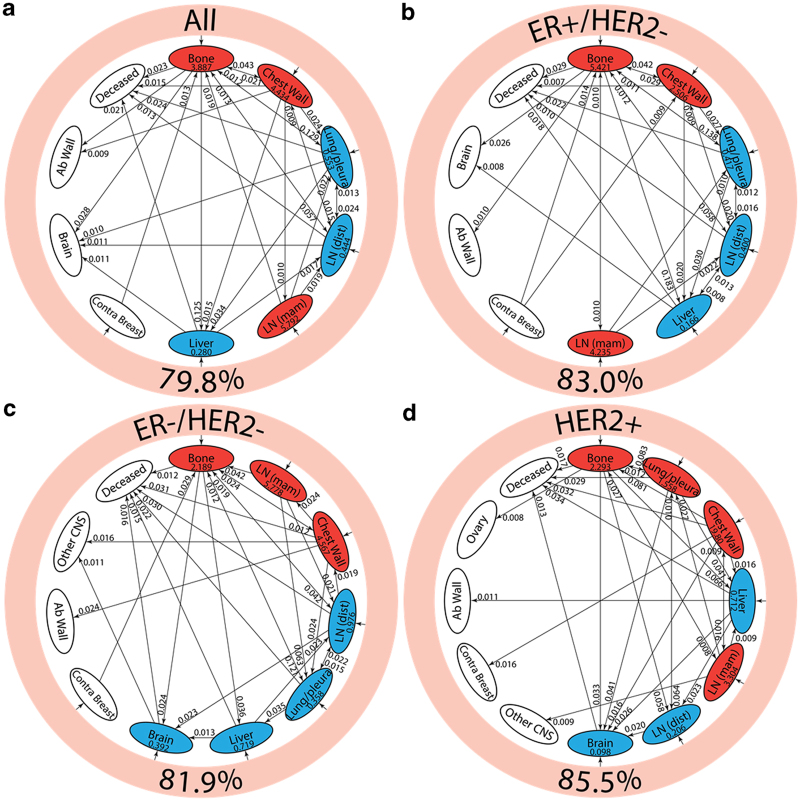
Pathway diagrams showing top 30 two-step pathways emanating from breast (pink ring). Nodes are classified as a “spreader” (red) or “sponge” (blue) based on the ratio of the incoming and outgoing two-step probabilities (spreader and sponge factor listed in respective ovals). (**a**) All patients’ pathway diagram representing 79.8% of total pathways, (**b**) ER+/HER2− pathway diagram representing 83.0% of total pathways, (**c**) ER−/HER2− pathway diagram representing 81.9% of total pathways, and (**d**) HER2+ pathway diagram representing 85.5% of total pathways.

Although the patterns of metastatic spread appear to be highly complex, they can be simplified by examination of respective components. The finding on Figure 3b showing that the pathways that breast cancer takes out of the breast is significantly less complex than the overall model in Figure 4a suggests that not only is the primary tumor a source for metastasis but also metastases themselves serve as sources of other metastatic sites and many of these secondary metastases are associated with high risk of transitioning to death as shown in Figure 3d.

**Figure 5:**
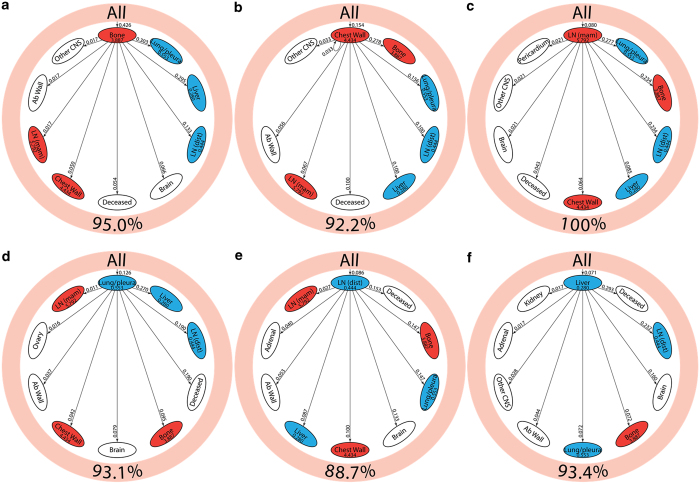
Spreader/sponge diagrams for all patients showing one-step transition probability from (**a**) bone, (**b**) chest wall, (**c**) LN (mam), (**d**) lung/pleura, (**e**) LN (dist), and (**f**) liver to the top nine sites in the network. Sites are ordered in decreasing order, clockwise, with the spreader/sponge in question located at 12:00. Outer pink ring represents primary breast cancer and shows the percentage of total transition probability it represents. dist, distant; LN, lymph node; mam, mammary.

**Figure 6:**
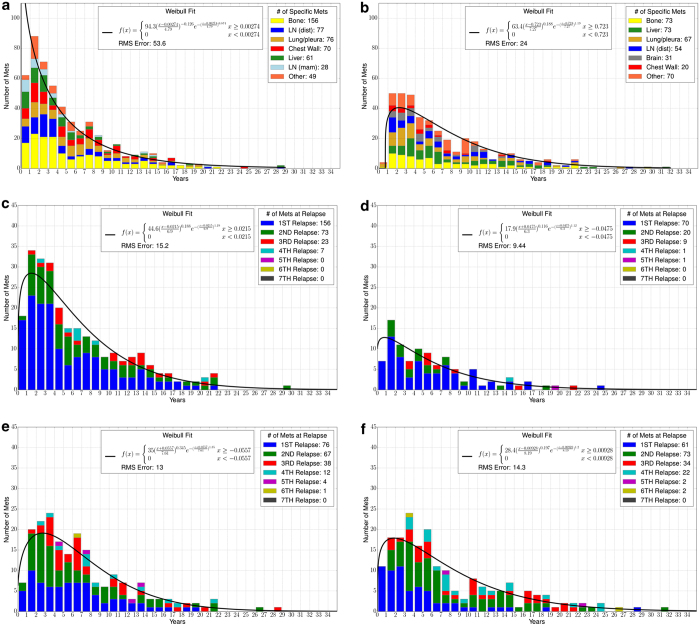
Histograms showing average time from diagnosis to (**a**) first metastatic site, (**b**) second metastatic site, (**c**) bone metastasis, (**d**) chest wall metastasis, (**e**) lung metastasis, and (**f**) liver metastasis. Graphs are color coded for specific metastases (**a** and **b**) or met relapse number (**c**–**f**). A two-parameter Weibull distribution is used as a curve fit.

Figure 4 shows the reduced spreader–sponge diagrams for all of the patients (Figure 4a), followed by diagrams for each of the subgroups ER+/HER2−, ER−/HER2−, HER2+. Sites colored in red are “spreader” sites, whose ratio of outgoing path probability to incoming path probability (called the amplification factor) is greater than one, whereas those colored blue are “sponge” sites, whose ratio of outgoing path probability to incoming path probability (called the absorption factor) is less than one. For all of the patients aggregated (Figure 4a), bone, chest wall, and mammary lymph nodes are the spreader sites, and lung/pleura, distant lymph nodes, and liver are the sponge sites. In Figure 4b we show the ER+/HER2− subgroup with the same spreader/sponge sites. The amplification factor of bone for this subgroup (5.421) is particularly high, whereas the absorption factor for liver (0.166) is quite low. For the ER−/HER2− group shown in Figure 4c, the mammary lymph nodes seem to be the strongest spreader (amplification factor of 5.778). Also of some significance is the brain in this subgroup becomes a sponge, with an absorption factor of 0.392. For the HER2+ subgroup shown in Figure 4d, lung/pleura and chest wall become the main spreaders, aside from bone. To clarify the spreader/sponge paths more clearly, Figure 5a–f shows the paths exiting from each of the main spreaders (all category), and each of the main sponges. Figure 6a,d, when viewed together, are instructive in that they show direct significant exchange between spreaders and sponges. For example, Figure 5a shows that lung/pleura is the most probable (sponge) site when exiting bone. Figure 5d, on the other hand, shows that cells that exit the lung/pleura site most probably go to liver, another sponge, but they can also go back to bone.

Prior animal and human experiments have supported the concept that metastatic sites are seeded and re-seeded with travel of cancer cells from one site to another via hematogenous routes;^20,21,28^ hence flow of cancer cells is likely to be bidirectional for all metastatic sites. However, the net flow will generally be in the direction from spreader sites to sponge sites. Although therapeutic interventions such as radiotherapy and hormonal therapy may have the ability to impact trafficking of tumor sites, the model did not incorporate differential effects of therapy into the model and the gradient of metastatic growth is modeled based on the population average and standard-of-care interventions. An understanding of the impact of therapeutics on the spreader and sponge characteristics of local sites requires further investigation.

### Metastatic relapse data

It is interesting to go further out in the pathway diagrams. Figure 1a shows that after developing a metastatic tumor at the bone site, the two most probable second metastatic sites are lung/pleura, and liver, both representing roughly equal sector widths (around 10%). But the future prognoses associated with those two groups of patients are quite different. In the case of the 49 patients who follow the breast–bone–lung path, the probability of transitioning to the “deceased” state is small (roughly 0.02). The highest next transition is to liver for this group (transition probability roughly 0.55). By contrast, the group who follow the breast–bone–liver pathway’s highest next transition probability is “deceased” (roughly 0.35), occurring on average roughly 2 years later. The most probable next transition for this group is to distant lymph nodes, with probability 0.31.

Of note, very few of these patients underwent pulmonary or hepatic resection or radiotherapy directed at a single organ site. We suspect that the more likely cause of this difference may be the spreader/sponge characteristics of these tumor types in metastatic breast cancer patients. If one examines subgroups, it is noted that for all groups except the triple-negative population, the liver is a more powerful sponge than the lungs, with smaller transition probabilities for liver than lung in the ER+ and/or HER2+ groups. It is within the ER+ and HER2+ groups that there is a low probability of transition from lung metastasis to death, whereas in the triple-negative group, where the transition probability is higher for liver than for lungs that we see an increased risk of death following development of a lung metastasis. Although in the absence of a second validation data set, it is possible that these findings are artifactual, they raise the possibility that organs that are more “sponge-like” relative to other affected organs are the anatomic sites at highest risk for organ failure leading to death. This is particularly true for visceral sites. Interestingly, regardless of immunohistochemistry characteristics of a given tumor or overall survival, all deceased patients died after an average of four metastatic sites.

### Temporal distributions

To link the discrete Markov time step “*k*” to the data, the temporal distributions are shown in the panel in Figure 6. Figure 6a shows the average time from diagnosis to first metastatic site, 5.30 years. The histogram is well modeled by a two-parameter Weibull distribution. Figure 6b shows the average time to the second metastatic site from diagnosis, 7.58 years. Figures 6c–f show the average times to a bone metastasis (6.53 years), chest wall metastasis (6.02 years), lung metastasis (6.92 years), and liver metastasis (7.72 years). All are well modeled by Weibull distributions. See the website http://kuhn.usc.edu/breast_cancer/ for temporal distributions associated with different subgroups.

## Discussion

Analyzing longitudinal data in terms of its combined spatiotemporal characteristics is an important step in building a comprehensive, robust, and predictive cancer progression model that can serve as a framework for statistical forecasting. There are several key points that are brought out in the current model worth reiterating. The first metastatic site very strongly influences future prognosis, even as much as ER/HER2 status of the patient. Second, the spreader–sponge classification of the metastatic sites is an important characteristic, with bone, mammary lymph nodes, and chest wall being the main spreaders for breast cancer, and distal lymph nodes, liver, and brain being sponges. However, the spreader–sponge character does depend on ER/HER2 status; the main example would be that lung/pleura are sponges for HER2−, but spreaders for HER2+. Whether or not this is because of differing treatments for these two groups is not clear.

Although most clinicians will, from experience, know that certain anatomic distributions of disease are associated with worse outcomes, the model presented here suggests that there is significant interplay between organ distribution and hormone-receptor status. Inclusion of therapeutics into the model, along with patient characteristics such as age, performance status, and genomic data has a potential to increase its complexity as well as predictive power. Although highly attuned physicians may have the capacity to replicate the predictive ability of the model, we anticipate that the performance of individual physicians to predict patient outcomes will be variable. For non-oncologists, a model such as this one may provide superior accuracy from the standpoint of prognosis. Moreover, noting that despite immunohistochemistry subgroups, nearly all patients approach death after four sites of metastatic disease may be clinically useful.

Additional efforts to refine the model in the future should include incorporation of therapeutic effects and the impact of prior therapy on the overall patterns of spread. Another limitation in the current model is an understanding of disease volume and the impact of disease volume of the course of patients. Refining the model to this higher level would require incorporation of raw radiography data. Although such complexity is beyond the current level of model development, future understanding of the role of disease volume in cancer spread is a necessary factor for future development.

The full data used to develop the model described in this paper are quite comprehensive and are available for graphical user-controlled viewing on the website: (http://kuhn.usc.edu/breast_cancer/).
